# Refractory iron deficiency anemia and Helicobacter Pylori Infection in pediatrics: A review

**Published:** 2015-03-15

**Authors:** Sh Gheibi, HR Farrokh-Eslamlou, M Noroozi, A Pakniyat

**Affiliations:** 1 Associate Professor of Pediatric Gastroenterology, Maternal and Childhood Obesity research Center, Urmia University of Medical Sciences, Shahid Motahari Hospital, Urmia, Iran. drgheibi@umsu.ac.ir; 2 Associate Professor of Maternal and Child Health, Reproductive Health Research Center, Urmia University of Medical Sciences. Urmia, I.R. Iran. hamidfarrokh@umsu.ac.ir; 3 Assistant Professor of Pediatric Hematology and Oncology, Department of Pediatric. Urmia University of Medical Sciences, I.R. Iran.; 4 Emergency medicine resident, Department of Emergency Medicine, Arak University of Medical Sciences , I.R Iran

**Keywords:** Iron deficiency anemia, Helicobacter Pylori Infection, pediatrics

## Abstract

**Background:**

Since the discovery of Helicobacter pylori, several clinical reports have demonstrated that H. Pylori infection has emerged as a new cause of refractory iron stores in children. We carried out a systematic literature review to primarily evaluate the existing evidence on the association between childhood H. Pylori infection and iron deficiency anemia (IDA) and secondly, to investigate the beneficial effects of bacterium elimination.

**Material and Methods:**

This review concerns important pediatric studies published from January 1991 to October 2014. Fourteen case reports and series of cases, 24 observational epidemiologic studies, seven uncontrolled trials, and 16 randomized clinical trials were included in the review.

**Results:**

Although there are a few observational epidemiologic studies and some randomized trials mostly due to the potential confounders, most studies reported a positive association linking between H. Pylori infection and iron deficiency or iron deficiency anemia among children. In addition, it seems that elimination of H. Pylori infection induces beneficial effects on iron deficiency.

**Conclusions:**

Since the evidence for the association of H. pylori eradication therapy and refractory childhood IDA is not enough and there are contrasting data about such association, future high quality and cohort researches are needed to determine the causal association.

## Introduction

Anemia, defined as a decrease in the amount of red blood cells (RBCs) or the amount of hemoglobin (Hb) concentration below established cut-off levels, is a global public health problem. Based on the World Health Organization (WHO) estimates, almost a quarter of the world`s population is anemic (1). Anemia occurs at all stages of the life cycle, but it is more prevalent in mothers and young children. It is estimated that 42% of pregnant women and 47% of preschool children are anemic (1). The most dramatic health consequence of severe anemia, which is well documented are increased risk of maternal and child mortality (2,3). Iron deficiency anemia caused the loss of 19.7 million disability-adjusted life years, accounting for 1.3% of the global total (4). Investigating the etiology of IDA in order to develop therapeutic strategies must be the first priority of health research agenda, especially in the developing countries. Many risk factors for IDA have been identified such as inadequate iron intake and absorption, increased iron requirements during growth, and excessive iron losses. In recent years, the association between Helicobacter Pylori (H. Pylori) infection and iron deficiency (ID) or IDA has been proposed seriously. 

H. Pylori is a highly prevalent microbial chronic infection across the world. Its worldwide prevalence is about 50% with a high variation related to the geography, age, and socioeconomic class. The overall prevalence is high in developing countries and lower in developed countries (5). It is estimated that 65% of children in developing countries are infected with H. pylori (6).In children, H. Pylori infection is associated with recurrent post-prandial abdominal pain, gastric dyspepsia, unexplained nausea and/or vomiting and duodenal ulcer which is the most known consequence of H. Pylori induced chronic gastritis and peptic ulcers (7). 

The association between H. pylori infection and IDA has been proposed since the last decade and currently attracted considerable interests (8, 9). H. pylori infection could cause IDA by several probable mechanisms: increasing iron loss due to active hemorrhage secondary to H. Pylori gastritis (10, 11), autoimmune atrophic gastritis (12), gastric cancer (13), reducing iron absorption following chronic pan-gastritis (14), and iron utilization by the bacterium (15). In addition, Low gastric acid secretion results in an impaired “gastric barrier,” which is associated with increased susceptibility to enteric infections, a major public health concern in the developing world (16).

Two meta-analyses concluded that H. pylori eradication therapy combining with or without iron administration is more effective than merely iron administration for IDA treatment (17, 18). Another meta-analysis conducted on Randomized Clinical Trials (RCTs), showed a non-significant improvement of Hb level following the eradication of H. pylori (19). Moreover, there is currently uncertainty regarding the effects of H. Pylori infection on IDA, especially sever one, and also of the H. Pylori eradication in the treatment of refractory IDA. Hence there is a need for a rigorous review to deal with aforementioned uncertainties. 

The aim of this study was to provide an extensive review of the available literature on decipher in order to the role of H. Pylori infection in IDA, especially in children age groups. 

## Materials and methods

**Types of studies**

Eligible studies were all publications, if they examined the associations of H. Pylori infection and IDA in children. We included all descriptive, case-control and cohort studies as well as RCTs, including cluster-randomized trials, quasi-randomized trials, and pre-post intervention studies in the current research. 

**Types of participants**

Those children below the age of 19 are eligible for the current study based on the United Nations Convention on the Rights of the Child which defines child as "a human being below the age of 18 years unless under the law applicable to the child, majority is attained earlier” (20).

Search methods for identification of studies

We included all English language computerized databases that are available to us in print or online. The full search strategies for MEDLINE and Pub Med Central are shown below.

#1: “Helicobacter Pylori” OR “Helicobacter Pylori infection” [MeSH]

#2: “anemia” [MeSH]

#3: “iron Deficiency” OR “iron” OR “nutritional anemia” OR “microcytic hypochromic anemia”[MeSH, all subheadings]

#4: “hemoglobin level” OR “hematocrit” OR “serum iron” OR “serum ferritin” OR “total iron binding capacity” [MeSH, all subheadings]

#5: #2 OR #3 OR #4

#6: (#1 AND #5)

**Electronic searches**

We searched the following computerized databases: Cochrane Central Register of Controlled Trials (CENTRAL), MEDLINE using Pub Med, Cumulative Index to Nursing and Allied Health (CINAHL), PMC using Pub Med, POPLINE, LILACS, Health STAR, and System for Information on Grey Literature in Europe (Open SIGLE). Additional references were retrieved from the option of “related articles” and the published reviews of this topic. The search was restricted to manuscripts published from January 1990 to 30 December 2014.The search results were grouped into three categories: case reports and case series on the association between H. pylori infection and ID or IDA among children, epidemiologic observational or sero-epidemiological studies of this association, and studies of response to treatment to eliminate HP infection with ID or IDA among children. Using a standard format, the following data were abstracted from each study: type of study, age and sex of the participants, study design and population, sample size, results of the study, and treatment group characteristics for the intervention trials. 

**Clinical evidences**

**Case reports and case series**

In 1991, the first evidence for an association between H. Pylori infection and serum iron in children was a case report which investigated a 15 years old girl suffering from anemia and concomitant H. Pylori associated chronic active hemorrhagic gastritis. Following the treatment of H. Pylori infection, her Hb values had become normal (21). The first evidence of association between H. Pylori infection and an unexplained IDA refractory to iron therapy came from another case report which described in a 7-year-old child without any gastrointestinal symptoms (22). In that patient, the refractory anemia had been attributed to the micronodular antritis due to a chronic non-active H. pylori related gastritis without hemorrhage. It also explained how the bacterium interfered with iron metabolism. Since then, several other case reports and series of cases have confirmed the relationship between H. Pylori infection and IDA among children and also presented refractory anemia cases with no apparent cause other than chronic H. Pylori-associated gastritis whose anemia was reversed only after H. pylori eradication without (23-29) or with (31-34) iron supplementation (Table 1).

**Observational epidemiologic studies**

During last 15 years, several sero-epidemiologic studies from both developed and developing countries demonstrated an association between H. pylori infection and decreasing iron status among children. Some population-based studies highlighted the association between H. Pylori infection and decreasing ferritin concentrations (35-42), whereas a number of them reported its association with decreasing level of ferritin and/or increasing prevalence of IDA among children (38, 41, 43-52) (Table 2). 

In an analysis of a cohort national survey of 1040 native children in Alaska, Parkinson et al. found a significant association between low serum ferritin levels and prevalence of H. pylori infection, particularly for child aged people (53). Bagget et al. confirmed the association of HP infection with ID and IDA among 688 children from the same population (45). Other large scale population-based studies from developed countries are listed in the followings: Sero-epidemiologic study on 1771 children in the USA showed an association between HP infections with increased risk for IDA (38). Muhsen et al. found a lower ferritin level among 509 under 19-year-old children (40) and also association of HP infection with higher prevalence of anemia in 399 under 9 year-old children (41). Fraser et al. studied 792 adolescents in New Zealand and found a positive association of HP infection with increased risk of ID, but not anemia (49). 

There are also several related observational epidemiologic studies in developing countries. Choe et al. conducted two sero-prevalence study among 660 (43) and 937 (36) school aged children in South Korea. They found that the relative risk of IDA was 2.9 (95% CI, 1.5 to 5.6) for those with HP infection and a predominant ID/IDA prevalence among H. pylori-positive girls, respectively. Kiran et al. from India examined 484 samples of 5-12 years children and declared a 19% prevalence of HP infection in anemic group and 10.7% in non-anemic group, which suggested an association between HP and IDA (50). Several sero-epidemiologic studies using smaller sample size from Taiwan (37), Iran (42) and Egypt (52, 54), pointed out association between HP infection and ID or IDA. 

Using upper gastrointestinal endoscopy, Süoglu et al. in Turkey (46) and Queiroz et al. in South America (55) revealed that H. Pylori infection was a significant predictor of IDA, low serum ferritin and hemoglobin concentrations among children. 

On the other hand, Santos et al. in a series of population-based multi-center and cross-sectional studies in Argentina, Bolivia, Mexico, and Venezuela, studied 2149 children aged between 4-17 and did not find any significant association between H. Pylori infection and anemia in children and adolescents (48). Also Araf et al. in Brazil (56), Vendt et al. (57), Choi et al. in South Korea (44), Sandström et al. in Sweden (58), and Zamani et al. in Iran (51) were not successful in finding any significant association between sero-prevalence of HP infection and the IDA. Whereas the role of H. pylori infection in unexplained IDA has already been confirmed by huge documents, this contradiction could be explained by the occurrence of some biases, such as the study design, H. pylori diagnosis criteria, and adjustment for confounding factors. 

**Clinical and interventional trials**

The most reliable evidence for a cause-and-effect comes from interventional trials which were identified for this review. Most of the identified studies showed that elimination of H. pylori associated with or without iron supplementation was followed by improvements in mean Hb levels, while others did not reveal such clear improvement in markers of iron deficiency (Table 3).

The first randomized controlled trial (RCT) to examine effects of H. Pylori treatment among children with refractory anemia without evidence of hemorrhage conducted by Choe et al. on 43 children with IDA in 1999 (59). They assigned 22 H. Pylori positive anemic girls to three groups: H. Pylori elimination treatment, iron supplementation only, and both elimination therapy plus iron supplementation. A significant increase in Hb level as compared with iron only group at 8 weeks after the therapy was found among patients who received eradication therapy (p = 0.0086). Choe et al gained the same result in another uncontrolled therapeutic trial on 13 patients (60). During recent 15 years, several controlled or uncontrolled trials conducted over diverse geographic areas in both developed and developing countries. For example, Ten RCTs have been conducted in China which all confirmed positive effect of H. Pylori eradication on the resolution of refractory IDA (61-70). Other studies from Italy, Japan, Greece, Turkey, and Mexico reported that complete recovery of IDA could be achieved with HP eradication with or without iron supplementation among children infected with H. Pylori (71-76).

The most interesting study is related to an un-blinded RCT conducted by Gessner et al. in Alaska, USA. The study population consisted of 219 children aged between 7-11 years having both ID and H. pylori infection (77). Briefly, patients were randomly assigned to two treatment groups: iron supplementation alone or iron supplementation with H. pylori elimination therapy. Iron status was reassessed at one, two, and 14 months after treatment ended. They reported that treatment of H. Pylori infection did not improve isolated ID or mild IDA up to 14 months after treatment initiation. Again the authors reassessed the same children 40 months after treatment and reported that the resolution of H. pylori infection for 40 months modestly reduced the prevalence of ID and substantially decreased the prevalence of IDA (78).

In line with Alaska, another interesting RCT conducted by Sarker et al. in an area highly endemic for ID and H. Pylori infection, in Bangladesh. They conducted a double-blind and placebo-controlled trial to evaluate four types of treatment of iron plus H. pylori therapy, H. pylori therapy and placebo, iron supplementation and placebo, and placebo alone on 200 children (79). After 90 days of follow-up, they concluded that H. Pylori infection was neither a cause of IDA/ID nor a reason for the treatment failure of iron. Recently, an uncontrolled trial on 18 school aged cases of iron deficient and H. Pylori infected in Saudi Arabia reported relatively the same result (80). They use H. Pylori treatment without iron supplementation and no significant relationship was found between eradication therapy and serum ferritin. 

A recent well designed double-blind RCT studied 110 non-anemic, asymptomatic and H. pylori-positive children (3-10 years old) in Texas, USA (81). The participants were randomly assigned to receive H. Pylori eradication therapy plus iron supplementation, iron supplementation only, or placebo. They concluded that H. Pylori infection eradication had a significant increasing effect on serum ferritin level.

The conflicting results of some of the above-mentioned trials could be attributed to the age and sex distribution of the cases due to the variation in physiologic iron loss and iron requirements, variation in the duration of post-treatment follow-up, and other trial design limitations such as small sample sizes. Such conflicting results found in the trials that examined the effect of H. Pylori elimination treatment on refractory IDA in children, were reflected in the findings of a few related meta-analyses. Based on the meta-analysis conducted by Qu et al., observational studies showed an association between H. pylori and IDA among children, but analysis of RCTs did not showed a significant improvement of Hemoglobin or ferritin levels following the eradication of H. pylori among IDA children (82). Huang et al. analyzed eight RCTs (four of which were conducted in children age groups) of H. pylori elimination and iron supplementation performed in Asia, an area with a high incidence of IDA and H pylori (17). They concluded that H. pylori eradication therapy combined with iron administration was more effective than iron administration alone for the treatment of refractory IDA.

**Table I:** Case reports and case series on the association between Helicobacter pylori (HP) infection and Iron Deficiency Anemia (IDA) among children

	**Cases**				
**Author**	n	Age (year)	Gender	Treatment	Results
**Blecker et al. 1991 (** 21 **)**	1	15	Female	HP eradication without Iron supplementation	Resolution of IDA
**Dufour et al. 1993 (** 22 **)**	1	7	Male	HP eradication without Iron supplementation	Resolution of IDA
**Bruel et al. 1993 (** 23 **)**	1	11	Unknown	HP eradication without Iron supplementation	Resolution of IDA
**Carnicer et al. 1997 (** 24 **)**	1	11	Female	HP eradication without Iron supplementation	Resolution of IDA
**Marignani et al. 1997 (** 25 **)**	1	School age	Unknown	HP eradication without Iron supplementation	Resolution of IDA
**Ashorn et al. 2001 (** 30 **)**	8	7-15	3 Female 5 Males	HP eradication with Iron supplementation	Resolution of IDA
**Nowicki et al. 2001 (** 31 **)**	1	12	Male	HP eradication with Iron supplementation	Resolution of IDA
**Sýkora et al. 2002 (** 26 **)**	1	15	Female	HP eradication without Iron supplementation	Resolution of IDA
					
**Hajikano et al. 2006 (** 27 **)**	1	6	Male	HP eradication without Iron supplementation	Resolution of IDA
**Cardamone et al. 2008 (** 28 **)**	3	10-14	1 Female 2 Males	HP eradication without Iron supplementation	Resolution of IDA
**Kotb et al. 2012 (** 32 **)**	20	8-18	? Female ? Males	HP eradication with Iron supplementation	Resolution of IDA
**Duclaux-Loras et al. 2013 (** 33 **)**	1	15	Male	HP eradication with Iron supplementation	Resolution of IDA
**Santalha et al. 2013 (** 29 **)**	1	12	Female	HP eradication without Iron supplementation	Resolution of IDA
**Miguel et al. 2014 (** 34 **)**	8	4.7-18	? Female ? Males	HP eradication with Iron supplementation (Four patients presented with HP infection object of eradication therapy.)	Resolution of IDA

**Table II:** Epidemiologic studies on the association between Helicobacter pylori (HP) infection and Iron Deficiency (ID) or Iron Deficiency Anemia (IDA) among children

		**Cases**		
**Study**	Type of study	n	Age (year)	Results
**Parkinson et al. 2000 (** 53 **) USA**	Population sero-prevalence	1040	child ages	A significant association between low serum ferritin levels and prevalence of HP infection found, particularly for children.
**Choe et al. 2001 (** 43 **)** **South Korea**	Population sero-prevalence	660	School age	The prevalence rates of HP -associated IDA in female athletes were higher than in the control group. The relative risk of IDA was 2.9 (95% CI, 1.5 to 5.6) for those with HP infection.
**Seo et al. 2002 (** 35 **)** **South Korea**	Population sero-prevalence	753	6-12	The prevalence of ID (ferritin < 15 ng/mL) in HP-seropositive children was significantly higher (13.9%) than in seronegative children (2.8%) and higher among girls. This association persisted after adjusting for age and their socioeconomic status (odds ratio, 5.6; 95% confidence interval, 1.0-30.6).
**Choe et al. 2003 (** 36 **)** **South Korea**	Population sero-prevalence	937	10-18	The HP positive rate in the IDA group was 44.8% in comparison with 20.0% in the non-IDA group (p=0.001). The serum ferritin level was significantly lower in the HP infected group (p=0.0002).
**Choi et al. 2003 (** 44 **)** **South Korea**	Population sero-prevalence	693	9-12	No significant differences in the sero-prevalence of HP infection and antibody titers to H. pylori were found between the IDA group and the non-anemic controls.
**Yang et al. 2005 (** 37 **) Taiwan**	Population sero-prevalence	163	1-16	HP infection in pre-adolescent children may determine iron deficiency and growth retardation.
**Baggett et al. 2006 (** 45 **) USA**	Population sero-prevalence	688	7-11	Active HP infection was independently associated with ID and IDA among children.
**Cardenas et al. 2006 (** 38 **) USA**	National Health	1771	≥3	HP infection was associated with the prevalence of IDA (prevalence odds ratio (POR) = 2.6, 95% CI: 1.5, 4.6) regardless of the presence or absence of peptic ulcer disease.
**DiGirolamo et al. 2007 (** 39 **) USA**	Population sero-prevalence	86	Mean 3.64	Presence of H. pylori antibodies emerged as a significant risk factor for anemia and iron deficiency in adjusted analyses controlling for demographic factors, current inflammation, and antibiotic use.
**Süoglu et al. 2007 (** 46 **) Turkey**	Hospital based study	70	4-16	Increased frequency of IDA in HP-infected patients in the present study supported similar findings in the literature.
**Haghi-Ashtiani et al. 2008 (** 47 **) Iran**	Case-Control	209	Median 7.1	The results did not support the proposal that HP infection was associated with IDA in children.
**Muhsen et al. 2009 (** 40 **)**	Population sero-prevalence	509	1-19	Low ferritin level was found between 14.5% and 8.6% of the HP seropositive and seronegative participants, respectively (P = 0.035).

**Table III:** Studies of response to treatment to eliminate HP infection with Iron Deficiency (ID) or Iron Deficiency Anemia (IDA) among children

Study	Study design	Age (Years)	Sample size	Treatment group	Results
Choe et al. 1999 (59) South Korea	RCT (Double-blind)	10-17	22 IDA cases, 18 controls	HP infection treatment with and without iron or placebo	Treatment of HP infection was associated with more rapid response to oral iron therapy as compared with the use of iron therapy alone.
Barabino et al. 1999 (71) Italy	Uncontrolled trial	4-14	4 IDA cases	HP infection treatment with iron supplementation	Resolution of IDA only after Iron supplementation.
Choe et al. 2000 (60) South Korea	Uncontrolled trial	15-17	13 IDA cases	HP infection treatment with iron supplementation	HP infection eradication along with iron supplementation could correct the refractory anemia among children.
Konno et al. 2000 (73) Japan	Uncontrolled trial	13-15	6IDA cases	HP infection treatment without iron supplementation	Resolution of IDA.
Guan et al. 2002 (61) China	RCT	13-19	14 IDA trial cases, 14 IDA controls	HP infection treatment with iron supplementation	Resolution of IDA.
Kostaki et al. 2003 (74)Greece	Uncontrolled trial	9-13	3 IDA cases	HP infection treatment with or without Iron supplementation	In all cases, long-standing iron supplementation became effective only after eradication of HP.
Russo-Mancuso et al. 2003 (72) Italy	Uncontrolled trial	4-18	9 unresponsive IDA cases	HP infection treatment with Iron supplementation	The eradication of HP was associated with stable normalization of iron stores.
Li et al. 2003 (62) China	RCT	14-19	21 IDA cases, 20 controls	HP infection treatment with Iron supplementation	Resolution of IDA.
Kurekci et al. 2005 (75) Turkey	Uncontrolled trial	6-16	18 IDA cases, 36 ID cases, 86 normal	HP infection treatment without iron supplementation	Complete recovery of ID and IDA could be achieved with HP eradication without iron supplementation in children with HP infection.
Lin et al. 2005 (63) China	RCT	6-12	35 IDA trial cases, 33 IDA controls	HP infection treatment with iron supplementation	Iron supplementation together with anti-HP therapy is more effective than iron supplementation therapy alone.
Wang et al. 2005 (64) China	RCT	10-18	30 IDA trial cases, 20 IDA controls	HP infection treatment with iron supplementation	Resolution of IDA.
Gessner et al. 2006 (77) USA	RCT (Un-blinded)	7-11	106 trial cases, 113 controls	HP infection treatment with iron supplementation	Treatment and resolution of HP infection did not improve isolated ID or mild IDA up to 14 months after treatment initiation.
Wang et al. 2006 (65) China	RCT	5-14	15 IDA trial cases, 14 IDA controls	HP infection treatment with iron supplementation	Resolution of IDA.

## Discussion

Since 1991 which the first evidence was published on the association between H. pylori infection and iron deficiency in children, there are many published related evidences which were case reports, observational epidemiologic studies, intervention trials and a very limited number of Mata-analyses.

We identified 14 case reports or series of cases that documented findings on the association between H. pylori infection and ID or IDA among children (21-34). Some of the case reports were highlighted the underlying pathologic causes of anemia in the cases, but most of those have confirmed the association between H. Pylori infection and IDA among children. Our review indicated that most of the reported refractory anemia cases among children were reversed only after H. pylori eradication without or with iron supplementation.

Our review identified 24 observational epidemiologic studies that reported findings associating H. Pylori and ID/IDA among children age groups in both developed and developing countries (35-58). However, a great variability was found across the observational epidemiologic studies, with regard to the differences in the geographical and ethnical distribution of children, age confounding, inclusion criteria, sample size, sampling procedures, methods of detecting anemia, and methods of detecting H. Pylori infection. In addition, there are two studies evaluating the association of H. pylori infection and ID/IDA in children undergoing upper gastrointestinal endoscopy (46, 55), which allow an accurate diagnosis of H. pylori infection. Except a few studies, the most of the sero-epidemiologic researches reported a significant association between reduced levels of iron stores in asymptomatic H. pylori-infected children, IDA and prevalence of HP infection found in children, although there are others that did not confirm the association (44, 47, 48, 58). This conclusion cannot be reliable, since in most of the studies, the applied design was cross sectional.

We found seven uncontrolled trials and 16 randomized clinical trials which are the most valuable demonstration of the beneficial effects of H. pylori infection eradication on pre-existing IDA in children (59-82). A great variability was found across the clinical trials, with regard to geographical, developmental and cultural distribution of children, study design, small sample size, sampling procedures, poor case definitions, methods of detecting anemia, lack of control groups, and methods of detecting H. Pylori infection and duration of follow-up. Eradication of H. pylori for refractory IDA is supported by most of the current evidences. However, larger sample and well designed RCTs are necessary to clarify the association between H. pylori infection and IDA among children.

## Conflict of Interest

There is no conflict of interest.
